# Improved Interlaminar Properties of Glass Fiber/Epoxy Laminates by the Synergic Modification of Soft and Rigid Particles

**DOI:** 10.3390/ma16196611

**Published:** 2023-10-09

**Authors:** Jingwei Liu, Shenghui Tian, Jiaqi Ren, Jin Huang, Lin Luo, Bing Du, Tianyong Zhang

**Affiliations:** 1 Chongqing Key Laboratory of Nano-Micro Composites and Devices, College of Metallurgy and Materials Engineering, Chongqing University of Science and Technology, Chongqing 401331, China; 2 Department of Fine Chemicals and Engineering, School of Chemical Engineering and Technology, Tianjin University, Tianjin 300072, China; tyzhang@tju.edu.cn; 3 Chongqing Key Laboratory of Soft Matter Materials Chemistry and Functional Manufacturing, School of Chemistry and Chemical Engineering, Southwest University, Chongqing 400715, China

**Keywords:** epoxy resin, carboxy-terminated nitrile butadiene rubber, nano-SiO_2_, toughening, GF/EP laminate composites, interlaminar properties

## Abstract

Poor interlaminar fracture toughness has been a major issue in glass fiber-reinforced epoxy resin (GF/EP) laminate composites. In this paper, soft carboxy-terminated nitrile (CTBN) rubber particles and rigid nano-SiO_2_ are used to toughen the epoxy resin (EP) matrix to improve the interlayer properties of GF/EP laminate composites. The effects of adding two toughening agents on the mechanical and interlayer properties of GF/EP laminates were studied. The results showed that adding the two kinds of particles improved the mechanical properties of the epoxy matrix. When the additional amount of flexible CTBN rubber particles was 8 wt%, and the rigid nano-SiO_2_ was 0.5 wt%, the fracture toughness of the matrix resin was increased by 215.8%, and the tensile strength was only decreased by 2.3% compared with the pure epoxy resin. On this basis, the effects of two kinds of particles on the interlayer properties of GF/EP composites were studied. Compared with the unmodified GF/EP laminates, the interlayer shear strength and mode I interlayer fracture toughness is significantly improved by a toughening agent, and the energy release rate *G_IC_* of interlayer shear strength and interlayer fracture toughness is increased by 109.2%, and 86.8%, respectively. The flexible CTBN rubber particles and rigid nano-SiO_2_ improve the interfacial adhesion between GF and EP. The cavitation of the two particles and the plastic deformation of the matrix is the toughening mechanism of the interlayer properties of the composite. Such excellent interlaminar mechanical properties make it possible for GF/EP laminates to be widely used as engineering materials in various industries (e.g., aerospace, hydrogen energy, marine).

## 1. Introduction

Glass fiber-reinforced epoxy resin (GF/EP) laminates have the advantages of high mechanical strength, stable size, electrical properties, strong designability, and low price. Thus, they are widely used in construction, electrical engineering, rail transit, and other fields [1,2]. However, the in-plane orientation of glass fibers makes the interlayer properties of GF/EP composites much lower than the in-plane properties [3,4]. Meanwhile, the difference in thermal expansion coefficient and Poisson’s ratio between the layers leads to intense stress concentration at the mechanical joints inside the composites. Weak interlaminar properties and severe stress concentration make GF/EP composites easy to produce minor cracks that are difficult to detect and expand into the layers under low-impact energy [5]. Gradually inducing interlaminar damage leads to the overall damage of the composite, which seriously hinders the application of GF/EP composites in key load-bearing components. Improving interlaminar properties in GF/EP composite laminates has attracted much attention over the past few decades [6,7].

According to the principle of material mechanics, there are two ideal modes of interface adhesive failure and cohesion failure in the layered failure process of fiber-reinforced composite materials [8], in which the external load energy absorbed by the interface adhesive failure process is much smaller than that absorbed by the cohesion failure process [9,10]. It is necessary to improve the interlayer bonding properties of composite materials and improve the failure work of matrix cohesion failure, which means toughening the matrix [11,12].

The modulus difference between the fiber and the matrix resin is significant, and the stress concentration at the interface can easily cause interface damage to the composite [5]. Li Gang et al. used organic molecules combined with inorganic nano-SiO_2_ particles to study the influence of matrix modulus changes on the interface bonding properties of high-modulus carbon fiber-reinforced epoxy resin composites. When the modulus of the epoxy matrix is increased by 30.6–34.4%, the interlaminar shear strength of its composite material is increased by 39.8–61.8%, and its failure mode changes from interface adhesive failure to matrix cohesion failure [13,14]. Similarly, LalLazar et al. added 0.75 wt% silicon dioxide (SiO_2_) with a particle size of 17 nm to the epoxy matrix [15]. Then, they combined it with an E-type glass fiber to prepare laminates, whose interlayer fracture toughness increased by 60% compared with that unmodified. Improving the stiffness matching between matrix and fiber by increasing the matrix modulus is an effective method to improve the interfacial stress transfer efficiency and enhance the interface bonding performance of the laminates.

For matrix toughening, the most prevalent toughening method is incorporating nano-sized or micro-sized fillers into epoxies. According to the different structures of the toughening modifier, it can be divided into polymer toughening [16] and rigid inorganic particle toughening [17,18,19]. Carboxylated-terminated acrylonitrile rubber (CTBN) is a polymer elastomer with active carboxyl functional groups at both ends of the molecular chain, and its glass transition temperature is much lower than room temperature. In curing with epoxy resin, the end carboxyl group of CTBN can participate in the crosslinking reaction of epoxy resin. With the curing of epoxy resin, phase separation occurs between the two polymers, and CTBN is dispersed in the epoxy resin matrix with rubber particles of different scales [20,21]. When the material is under load, the curing residual stress of the particle and the three-dimensional stress at the crack front are superimposed, which leads to the interface debonding between the particles and the matrix. The interface debonding generates many voids in the matrix, triggering the shear band of the nearby matrix, which ultimately consumes the applied load and prevents the crack propagation [22,23]. CTBN toughening epoxy resin has the advantage of high efficiency. Adding 10~40 wt% CTBN can increase the toughness of epoxy resin by several times or even more than ten times [24]. However, the addition of elastomers also causes the strength of epoxy resin to diminish, the glass transition temperature to drop, and the thermal expansion coefficient to rise [24,25].

For interlaminar toughening, it has been demonstrated that rubber frequently encourages inherent and extrinsic energy dissipation mechanisms like cavitation and matrix yielding that prevent the interlaminar crack from propagating. About 5~20 wt% rubber can increase the interlaminar toughness of fiber-reinforced epoxy resin by 52~85% [26,27,28,29]. More recently, some authors [30,31] investigated the use of rubber nanofibers, such as nitrile butadiene rubber/poly(ε-caprolactone)-blend nanofibers [31], producing a similar interlayer toughening effect while having far better damping qualities. However, adding nanofibers is expensive, and they should only be utilized to reinforce particular areas that experience severe mechanical loads, not to change the bulk resin [32].

Rigid inorganic particles with high modulus and chemical stability are commonly used as fillers in epoxy composites [33]. The composite of nano-SiO_2_ with the epoxy matrix can significantly increase the matrix modulus [34,35,36] and enhance the interface between the fiber phase and matrix phase. In addition, the fracture failure work of the interlayer matrix phase can be increased by crack deflection, interface debonding, and matrix plastic deformation, which is a hot spot in the field of interlayer toughening of fiber-reinforced composites [37]. Studies have shown that adding 1~20 wt% nano-SiO_2_ to epoxy resin can increase the resin’s elastic modulus and fracture toughness by 5–40% and 10–52%, respectively. However, due to the huge specific surface area of nanomaterials, the viscosity of the epoxy matrix will increase, which is not conducive to the processing performance of composite materials [38].

In summary, the soft CTBN rubber particles are conducive to the improvement of the fracture toughness of the epoxy resin matrix. Nevertheless, the flexibility of the rubber also brings about the problem of reducing the modulus and heat resistance of the epoxy resin, which affects the interface strength of GF/EP. However, rigid nano-SiO_2_ can improve the strength and modulus of epoxy resin and then enhance the interface failure strength by reducing the difference in the modulus of the GF/EP interface [39]. Based on these conclusions, this paper used soft CTBN rubber particles and rigid nano-SiO_2_ as co-toughening agents to modify the epoxy matrix. Then, the co-toughened epoxy matrix was hot-pressed with glass fiber to prepare GF/EP laminates. The effects of rigid and soft nanoparticles on the interlayer properties of laminates were studied. The fracture process of GF/EP-laminated composites was investigated using sectional analysis. In this study, the correlation between matrix modulus and interlaminar damage process is confirmed. The modulus of the matrix is preserved while the matrix is effectively toughened thanks to the synergistic action of soft and hard particles. The GF/EP laminates’ interlaminar characteristics were greatly enhanced. The materials used in the study are all industrialized and easy to industrialize mass production. This article provides theoretical and technical guidance for the industrial manufacturing of high-performance GF/EP laminates.

## 2. Experimental Section

### 2.1. Reagents

Bisphenol A type (DGEBA) epoxy resin (CYD-128, epoxy value 0.51, density 1.22 g/cm^3^, purity > 95%), purchased from Guangzhou Nasun Chemical Co., Ltd., Guangzhou, China. Methyl-tetrahydro phthalic anhydride (MTHPA, purity > 98%), purchased from Kunshan Lvdun Chemical Co., Ltd., Suzhou, China. N, N-dimethylbenzylamine (purity > 98%) purchased from Shanghai Sinopsin Group Chemical Reagent Co., Ltd., Shanghai, China. CTBN (industrial grade) was purchased from Yuexin Chemical Plastics firm, Tianjin, China. Nano-SiO_2_ (100 nm, purity > 98%) purchased from Boas Nanotechnology (Ningbo) Co., Ltd., Ningbo, China.

### 2.2. Preparation of Epoxy Matrix Modified by CTBN

CTBN rubber is added to E-51 epoxy resin in proportion, with MTHPA as the curing agent and N, N-dimethylbenzylamine as the curing accelerator. The ratios described in this paper are all by weight. The specific steps are as follows:

1. Approximately 5 wt%, 8 wt%, and 10 wt% CTBN were added into the unmodified resin matrix based on epoxy resin, respectively.
2. The unmodified and modified resin matrix were prepared with the weight ratio of epoxy resin:MTHPA:N, N-dimethylbenzylamine = 100:90:2.
3. The mixture is placed in the rotation agitator (Thinky Mixer ARE-310) to be stirred and defoamed.
4. The stirred liquid was slowly poured into the mold and put in the oven at 60 °C for vacuum defoaming for 30 min.
5. The mold is placed into the oven for curing, and the procedure is conducted at 80 °C for 4 h, and then at 140 °C for 8 h.

### 2.3. Preparation of Resin Matrix with Synergic Toughening of Nano-SiO_2_ and CTBN

1. Based on epoxy resin, CTBN with the optimal ratio was added, and nano-SiO_2_ with the additional amount of 0.25 wt%, 0.5 wt%, 0.75 wt%, and 1.0 wt% were added to tetrahydrofuran. Ultrasonic dispersion was carried out for 30 min.
2. Corresponding quality epoxy resin was added into the tetrahydrofuran nano-SiO_2_ solution and stirred for 5 min.
3. The tetrahydrofuran in the resin was removed successively by rotating the evaporation apparatus and vacuum oven. The curing agent and accelerator were added at the ratio of epoxy resin:MTHPA:N, N-dimethylbenzylamine = 100:90:2. For the following steps, refer to Section 2.2.

### 2.4. The Preparation of GF/EP Laminate Composites Toughened by Nano-SiO_2_ and CTBN 

**Figure 1:**
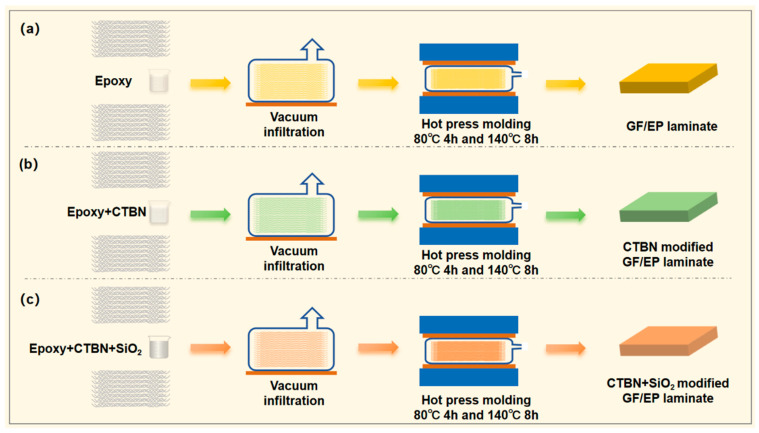
Schematic for the fabrication of GF/EP laminates (**a**) and CTBN- (**b**) or CTBN + SiO_2_- (**c**) modified GF/EP laminates.

The preparation requirements of the specimens for testing the fracture toughness and shear strength of mode I are different, and the preparation process is slightly different. The preparation of GF/EP laminates with mode I fracture toughness was taken as an example. The specific operation steps are described below and schematically shown in Figure 1.

1. The glass fiber cloth was cut into a rectangular fabric of the required size. The glass fiber cloth was washed with ethanol absolute (ethanol concentration ≥ 99.5%) and put into the oven for drying.
2. According to the configuration of the resin matrix, the epoxy resin matrix with different components of nano-SiO_2_ and CTBN is prepared.
3. Use a brush dipped in a small amount of glue to coat the glass fiber cloth, use a scraper to scrape off excess resin after coating, and then lay the 2nd~12th layer in turn.
4. After the 12th layer is laid, spread polyimide with a thickness of 25 μm in the width direction as a prefab crack (this step is unnecessary for the preparation of shear strength laminate composite materials), and continue to apply the adhesive liquid on the remaining glass fiber cloth. The layering of the 13th to 24th layers is the same as layers 2nd to 12.
5. After the 24th layer is covered, the system is put into a vacuum bag to vacuum and remove bubbles for 30 min. Put the system into the mold and use the hot press for hot pressing. The curing procedure is 80 °C 4 h and 140 °C 8 h. The thickness of the laminates was about 3 mm, with the fiber volume fraction of 60~65%.

### 2.5. Test and Characterization

#### 2.5.1. Fracture Testing

The fracture toughness of the epoxy bulk materials was tested based on the single-edge-notch bend (SENB) sample following ASTMD 5045-14 [40]. Standard samples with dimensions of 70 mm, 10 mm, and 4 mm in length, width, and thickness, respectively, were made using molds. The saw blade was used to process a 1 mm notch in the middle of the spline where there was no bubble, and then the blade was used to form a natural crack with a length of 4.5~5.5 mm inside the specimens. After annealing at 60 °C, a universal testing machine was used to carry out a three-point bending test on the treated specimens. The specimens were stored and tested at a standard laboratory atmosphere of 23 ± 3 °C and 50 ± 10% relative humidity. Five samples were tested for each material group. The span was 40 mm, and the loading speed was 1 mm/min. The sample thickness *B* (mm) and width *W* (mm), crack length *a* (mm), and maximum pressure *P_max_* (N) of the spline were recorded after the test. The critical stress intensity factor (*K_IC_*, MPa.m^1/2^) of the sample was calculated according to the following formula:

(1) $$K_{IC}=\left(\frac{P_{max}}{{BW}^{1/2}}\right)f\left(x\right)$$

where *x* = *a*/*W* (0 < *x* < 1), *f*(*x*) is the geometric correction factor related to crack length, calculated using Equation (2):

(2) $$f(x)=6x^{1/2}\frac{1.99-x(1-x)(2.15-3.93x+2.7x^{2})}{(1+2x)(1-x{)}^{3/2}}$$

According to the standard ASTMD 5528 [41], the double cantilever beam (DCB) specimen was used. The specimen’s length and width (*b*) were 150 mm and 20 mm, respectively. The prefabricated crack length was about 35 mm. Smooth each edge of the cut spline, and measure the actual width of the spline *b* (mm). The hinges were glued on both sides of the spline and placed at room temperature for 24 h. Specimens were stored and tested at a standard laboratory atmosphere of 23 ± 3 °C and 50 ± 10% relative humidity. The universal testing machine program was set to the tensile mode, with a 2 mm/min loading rate. When the sample was peeled 50 mm from the start of the prefabricated crack, the displacement *δ* (mm) and the load *P* (N) were recorded for each 5 mm crack length *a* (mm) extended. When the sample was peeled entirely, the test ended. The relationship between the energy release rate of interlayer peel toughness and the crack length can be obtained through the load–displacement curve, and the crack length is read synchronously online. The propagation interlaminar fracture energy (*G_IC_*_,*Pro*_) was the average *G_IC_* value when the crack growth (Δ*a*) was increased from 15 to 65 mm. Then, the *G_IC,Pro_* value of the mode I fracture toughness of the laminated composite with or without CTBN and nano-SiO_2_ can be obtained. Five samples were tested for each material system and the average result was recorded.

(3) $$G_{IC}=\frac{3P\delta }{2ab}$$

#### 2.5.2. Tensile Property Testing

According to GB/T 2567-2021 [42], the unmodified and modified epoxy matrix materials were poured into dumbbell splines with a length of 200 mm, in which the narrow side was 60 mm long, 10 mm wide, and 4 mm thick. Specimens were stored and tested at a standard laboratory atmosphere of 23 ± 3 °C and 50 ± 10% relative humidity. The universal testing machine was used to test the treated splines at a 2 mm/min loading speed. The maximum stress of material failure was taken as the tensile strength, and more than 5 effective values were divided into each group, and their average values were taken.

#### 2.5.3. SEM Observation of Section Morphology

After the fracture toughness and tensile test of the resin spline, the sample was fixed on the copper sample table with conductive adhesive. Platinum was sprayed twice under a vacuum for observation, and the accelerated voltage was 5 KV.

#### 2.5.4. GF/EP Laminates Interlayer Shear Strength Test

According to standard JC/T773 [43], the laminated composites were cut into specimens with dimensions of 30 mm and 15 mm, respectively, and each edge was polished flat. The spline’s actual width *b* (mm) and thickness *h* (mm) were recorded. The universal testing machine performed a three-point bending test on the spline. The span was 15 mm, and the loading rate was 1 mm/min. The maximum load *F* (N) carried by the sample was recorded. The interlaminar shear strength of the laminated composite was calculated according to Equation (3). The specimens were stored and tested at a standard laboratory atmosphere of 23 ± 3 °C and 50 ± 10% relative humidity. At least three samples were tested for each material system and the average result was recorded.

(4) $$\tau_{m}=\frac{3F}{4bh}$$

## 3. Results and Discussion

### 3.1. Mechanical Properties of Epoxy Matrix Modified by Particles

**Figure 2:**
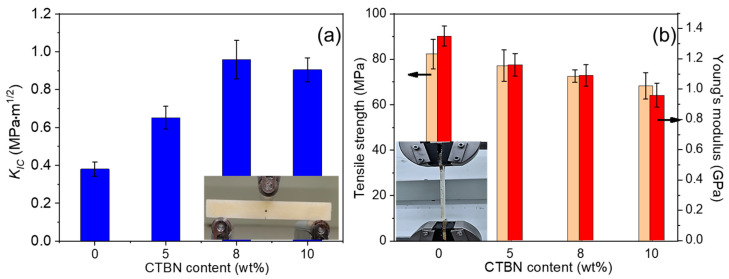
Critical stress intensity factor (*K_IC_*) (**a**) and tensile strength (**b**) of CTBN-modified epoxy resin matrix.

The mode I fracture toughness and tensile properties of epoxy resin were tested, respectively. As shown in Figure 2a, with the increase in CTBN addition, the epoxy resin matrix’s *K_IC_* value showed an increasing trend. The *K_IC_* value of pure epoxy resin was 0.38 MPa.m^1/2^, and after adding 5 wt%, 8 wt%, and 10 wt% CTBN, the *K_IC_* value was 0.65 MPa.m^1/2^, 0.95 MPa.m^1/2^, and 0.90 MPa.m^1/2^, respectively, which showed an increase in value by 71%, 150%, and 137%, respectively, compared with pure epoxy resin. It can be seen that the addition of the CTBN toughening agent can improve the toughness of epoxy resin. When the amount added is 8 wt%, the fracture toughness of the epoxy matrix is the best, and the continued addition will lead to the slight decline of toughness. Since the toughening mechanism of epoxy by submicron rubber particles is mainly to produce holes and matrix plastic shear, which weakens the stress field at the crack tip, when the number of particles inside the material reaches the peak, the addition of CTBN cannot produce more holes or matrix plastic yield and also reduces the efficiency of internal stress transfer. Therefore, as the CTBN loading continues to increase, the fracture toughness of the composites begins to decrease.

As shown in Figure 2b, the tensile strength of epoxy resin decreased with the increase in CTBN addition. The tensile strength of pure epoxy resin is 82.3 MPa. When 5 wt%, 8 wt%, and 10 wt% CTBN were added, the tensile strength was 77.2 MPa, 72.6 MPa, and 68.3 MPa, respectively, which decreased by 6.2%, 11.8%, and 17.0% compared with pure epoxy resin, respectively. The strength decrease is attributed to the reduced cohesion energy density of the epoxy by the addition of flexible CTBN particles. The best compromise approach combines the fracture toughness of CTBN-modified epoxy resin matrix with epoxy resin by adding 8 wt% of CTBN. The epoxy composite currently has the highest fracture toughness (0.95 MPa.m^1/2^), but its tensile strength is declining by 11.8%. The modulus reduction upon adding the CTBN was expected since the particles contained soft polymer polybutadiene, and its modulus was considerably lower than that of the epoxy resins [11]. For the neat epoxy, the Young’s modulus was 1.35 GPa. At 8 wt% CTBN, the epoxy matrix Young’s modulus showed a 19.3% decrease relative to the neat epoxy.

The authors tried to reduce the influence of rubber particles on the strength of the epoxy matrix. SiO_2_ nanoparticles were added to the epoxy resin toughened by 8 wt% CTBN for collaborative toughening. As shown in Figure 2a, in the epoxy resin matrix containing 8 wt% CTBN, the fracture toughness K_IC_ value of the material first increased and then reached the plateau with the loading of nano-SiO_2_ up to 0.5~1.0 wt%. When nano-SiO_2_ is not added, the *K_IC_* value of 8 wt% CTBN epoxy resin is 0.95 MPa.m^1/2^. When 0.25 wt%, 0.5 wt%, 0.75 wt%, and 1 wt% nano-SiO_2_ were added, the *K_IC_* values of the modified epoxy matrix were 0.99 MPa.m^1/2^, 1.20 MPa.m^1/2^, 1.26 MPa.m^1/2^, and 1.22 MPa.m^1/2^, respectively. Compared with the matrix modified by 8 wt% CTBN, the increases were 4.2%, 26.3%, 32.6%, and 28.4%, respectively. Adding nano-SiO_2_ further improves the fracture toughness of the epoxy resin matrix. When 0.75 wt% nano-SiO_2_ is added, the toughness of the epoxy resin matrix is the best, and the fracture toughness is 131.6% higher than that of the unmodified epoxy matrix.

**Figure 3:**
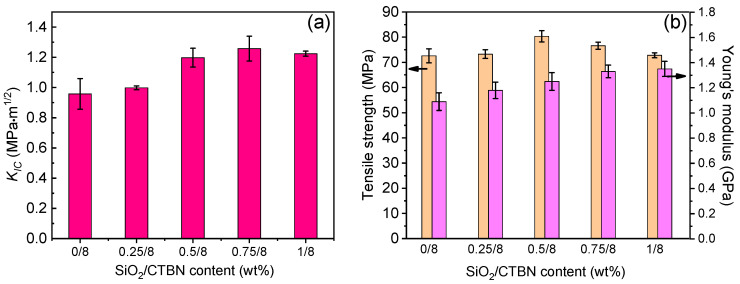
Critical stress intensity factor (*K_IC_*) (**a**) and tensile strength (**b**) of epoxy resin matrix co-modified by CTBN and nano-SiO_2_.

Figure 3b shows the effect of nano-SiO_2_ on the tensile strength of 8 wt% CTBN-modified matrix. As shown from the figure, the addition of nano-SiO_2_ causes the tensile strength of the epoxy matrix modified by 8 wt% CTBN to first increase and then decrease. The tensile strength of the epoxy matrix modified by 8 wt% CTBN is 72.6 MPa, and the addition of 0.25 wt%, 0.5 wt%, 0.75 wt%, and 1 wt% nano-SiO_2_. The tensile strength of the epoxy matrix modified by two-component particles is 73.3 MPa, 80.4 MPa, 76.6 MPa, and 73.8 MPa, respectively. The nano-SiO_2_ concentration was found to be optimal at 0.5 wt%, resulting in a 10.7% increase in tensile strength relative to the one-component-modified matrix. Compared with the unmodified epoxy matrix, the fracture toughness of the matrix is only 2.3% lower, and the fracture toughness is 215.8% higher than that of the unmodified epoxy matrix. Meanwhile, it can be seen that the introduction of nano-SiO_2_ dramatically improved the matrix in stiffness. For the 8 wt% CTBN-modified epoxy, the Young’s modulus is 1.09 GPa. At 0.25 wt%, 0.5 wt%, 0.75 wt%, and 1 wt% nano-SiO_2_, the Young’s modulus of matrix is 1.18 GPa, 1.25 GPa, 1.33 GPa, and 1.35 GPa, corresponding to the increases of 8.2%, 14.7%, 22%, and 23.9%, respectively. The Young’s modulus of epoxy matrix slightly reduces as the concentration of nano-SiO_2_ reaches 0.5 wt% (1.25 GPa) comparing with neat epoxy (1.35 GPa).

Considering the matrix’s fracture toughness and tensile properties, the synergistic modification of 0.5 wt% nano-SiO_2_ and 8 wt% CTBN rubber particles has a balanced strengthening and toughening effect on the epoxy matrix.

**Figure 4:**
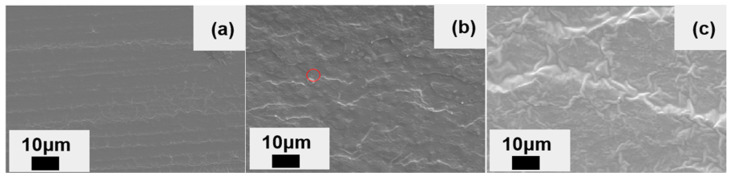
SEM image of epoxy resin matrix I toughness test section with unmodified (**a**), 8 wt% CTBN (**b**), and 8 wt% CTBN + 0.5 wt% nanoSiO_2_ (**c**).

Figure 4 shows the SEM images of the SENB specimen ductile section of pure epoxy, single-component CTBN modification, and the two-component co-modification of CTBN and nano-SiO_2_. As can be seen from Figure 4a, the surface of the mode I fracture toughness section of pure epoxy resin is smooth and flat, and there are relatively regular parallel banded lines, indicating that cracks expand faster inside the material without crack deflection or bifurcation, which is a typical brittle fracture feature. As shown in Figure 4b, the 8 wt% CTBN-toughened epoxy matrix mode I fracture toughness section has multiple curved and bifurcated river-like lines with a certain depth. The curved and bifurcated river-like lines indicate that cracks have experienced more crack deflection or bifurcation during the propagation process and have a longer propagation path, thereby improving the toughness of the resin. With the introduction of nanoparticles (Figure 4c), there were more bifurcated and curved lines on the surface of the section. There were wrinkle-like protrusions, indicating that the introduction of nanoparticles extended the propagation path of cracks in the epoxy resin before failure and caused a certain degree of plastic shear deformation of the epoxy, which was consistent with the further increase in the fracture toughness of the matrix material.

### 3.2. Interlayer Properties of GF/EP-Laminated Composites Toughened by SiO_2_ and CTBN

**Figure 5:**
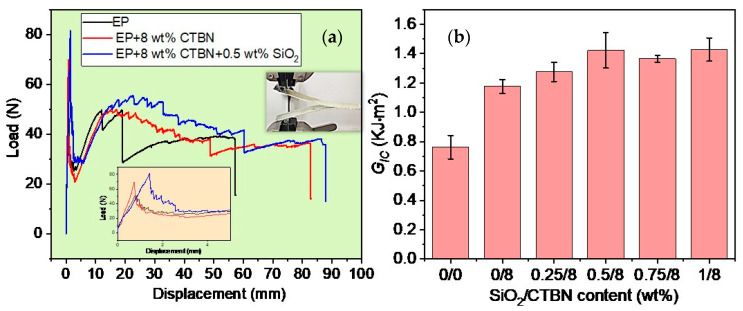
Typical load–displacement curves (**a**) of GF/EP-laminated composites with or without CTBN or SiO_2_ in the mode I interlayer stripping experiment and the average value of the calculated *G_IC_*_,*Pro*_ value (**b**).

(1) Interlayer fracture toughness: Figure 5a shows the typical load–displacement curve obtained in the mode I interlayer stripping experiment of GF/EP laminates with or without CTBN or SiO_2_ added. It can be seen that the load–displacement curve (black line) of the DCB specimen of unmodified GF/EP laminate composite material has two significant steps. The rest of the curve is relatively smooth. We analyze the reasons as follows. Under mechanical stress, the brittle epoxy cannot adequately dissipate the mechanical energy, resulting in crack propagation. These cracks propagate easily and quickly to a certain point, leading to a load drop. When 8 wt% CTBN is added, the load–displacement curve (red line) of the DCB specimen is in zigzags, meaning that the cavitation of rubber particles and the plastic deformation of the matrix material are obstructed in the crack propagation, and the displacement load and total displacement of the initial crack increase significantly. On this basis, when 0.5 wt% SiO_2_ is added, the total displacement of the load exceeds 85 mm, and the area under the load–displacement curve is much larger than that of the GF/EP composite DCB specimen without the modifier.

Through the load–displacement curve and the crack length, which reads synchronously online, the relationship between the energy release rate of interlayer peel toughness and the crack length change can be obtained, and then the *G_IC_*_,*Pro*_ value of the mode I fracture toughness of the laminated composite with or without CTBN and nano-SiO_2_ added can be obtained (as shown in Figure 5b).

Unmodified GF/EP laminates have an energy release rate with a *G_IC_*_,*Pro*_ value of 0.76 kJ/m^2^, and when 8 wt% CTBN is added, the *G_IC_*_,*Pro*_ value increases to 1.18 kJ/m^2^, which is 55.3% higher than unmodified GF/EP laminates. Furthermore, the laminates’ *G_IC_*_,*Pro*_ values rose to 1.28 kJ/m^2^, 1.42 kJ/m^2^, 1.37 kJ/m^2^, and 1.43 kJ/m^2^ when 0.25 wt%, 0.5 wt%, 0.75 wt%, and 1 wt% nano-SiO_2_ were added, respectively, to the 8 wt% CTBN-toughened EP system. The *G_IC_*_,*Pro*_ value of the laminated composite increased by 86.8% when 0.5 wt% nano-SiO_2_ was introduced, compared to the *G_IC_*_,*Pro*_ value of unmodified GF/EP-laminated composite.

**Figure 6:**
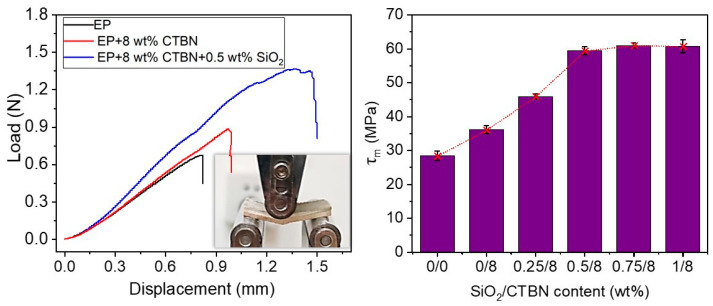
Typical load–displacement curves (**left**) of GF/EP-laminated composites with or without CTBN or SiO_2_ in the shear strength experiment and the saverage value (**right**) of the calculated shear strength of GF/EP-laminated composite toughened by SiO_2_ and CTBN.

(2) Delamination in fiber-reinforced resin composites is mostly caused by interlaminar shear forces. Figure 6 displays the shear strength of GF/EP-laminated composites. The shear strength of GF/EP laminate composites are greatly enhanced by CTBN rubber particles and nano-SiO_2_. Unmodified GF/EP has a shear strength of 28.4 MPa, and when 8 wt% CTBN is added, it has a shear strength of 36.1 MPa, which is 27.1% higher than that of unmodified GF/EP. The shear strength of the laminate increases dramatically when 0.25 wt% nano-SiO_2_ is added to 8 wt% CTBN-toughened GF/EP laminate, reaching 45.9 MPa, which is 27.1% higher than that of the single-component CTBN-modified GF/EP laminate. The interlayer shear strength of the laminate improves as the nano-SiO_2_ loading rises to 0.5 wt% (the slope of the red dashed line rises in Figure 6), reaching 59.4 MPa, which is 64.5% higher than that of the single-component-modified GF/EP laminate and 109.2% higher than that of the unmodified GF/EP laminate. When the loading of nano-SiO_2_ added to the 8% CTBN-toughened epoxy was raised from 0.5 wt% to 0.75 wt%, the improvement in the interlaminar shear strength of the composites slowed (in Figure 6, the slope of the red dotted line drops). The interlayer shear strength of the two-component-modified GF/EP laminate is optimum at 0.75 wt% nano-SiO_2_ introduction. The toughened GF/EP-laminated composite is 114% stronger than the unmodified GF/EP-laminated composite and 69.1% stronger than single-component CTBN.

### 3.3. Fracture Behavior Analysis of GF/EP Laminate Composites Toughened by Nano-SiO_2_ and CTBN 

**Figure 7:**
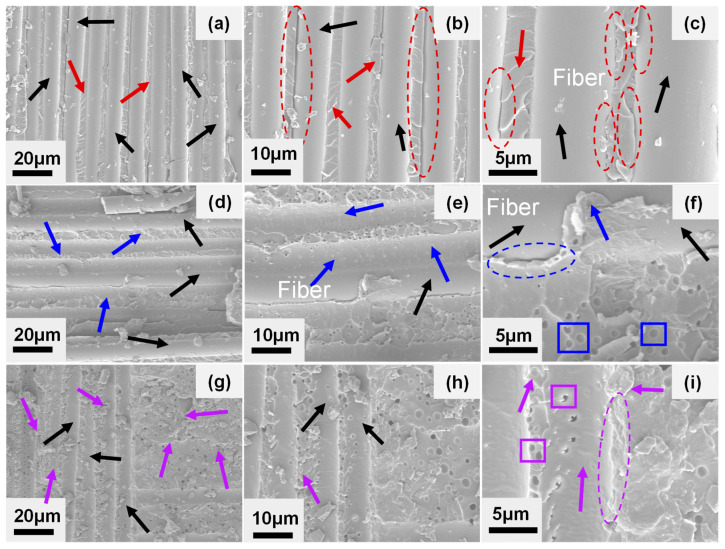
SEM image of mode I fracture toughness test (DCB specimen) of GF/EP-laminated composite material, which is not modified (**a**–**c**), modified (**d**–**f**) by 8 wt% CTBN, and co-modified (**g**–**i**) by 0.5 wt% nano-SiO_2_ + 8 wt% CTBN.

The fracture surface analysis is helpful to analyze the fracture behavior of materials [44]. Figure 7 shows the SEM topography of the GF/EP laminates’ DCB specimen with different components, and the crack propagation direction is from bottom to top. Figure 7a–c show the section morphology of the DCB specimen of unmodified GF/EP laminate. It can be seen from the figure that the glass fiber (black arrow) with delamination failure has a smooth surface and no matrix adhesion (as shown in Figure 7c), and there is a resin matrix (red arrow) with parallel river-like lines between adjacent glass fibers (black arrow). These characteristics indicate that the interface bonding between the unmodified epoxy matrix and the glass fiber is weak, and the cracks spread rapidly along the interface between the matrix and the fiber before failure. The failure mode belonged mainly to the interface debonding failure [45], so its *G_IC_*_,*Pro*_ value is low, only 0.76 kJ/m^2^.

Figure 7d–f shows the SEM cross-section of the DCB specimen of GF/EP laminates modified by 8 wt% CTBN. It can be seen from the figure that the surface of the glass fiber is partially covered with a thin matrix (blue arrow in Figure 7e,f). In contrast, some glass fibers have smooth surfaces without matrix adhesion, indicating that cracks are spreading in the matrix. It is a mixed failure mode in which matrix cohesion failure and interface adhesive failure coexist. At this time, the surface of the matrix (blue arrow) between the parallel glass fibers is rougher than the surface of the unmodified matrix, with more irregular mountain ridges. There are uniformly distributed voids [32,46] (blue box) in the enlarged figure (Figure 7e,f), which indicates that when the cracks expand in the matrix, the rubber particles will be hollow and cause the plastic deformation of the nearby matrix.

The coexisting failure mode of cohesion failure and interface adhesive failure and the increased matrix plastic deformation makes the crack propagation in GF/EP laminate composite increase in load consumption, wherein the interlaminar fracture toughness is significantly improved, and the *G_IC_*_,*Pro*_ value increases to 1.18 kJ/m^2^. It is worth noting that due to the negative effect of CTBN rubber particles on the modulus of the epoxy matrix, the stress transfer efficiency between the fiber and the matrix decreases, and part of the interface debonding also occurs under load (the exposed glass fiber and blue dotted elliptic crack in Figure 7f).

After 0.5 wt% nano-SiO_2_ was added to the epoxy matrix modified by 8 wt% CTBN, the glass fiber surface of the DCB specimen of GF/EP composite was covered with the matrix (black and purple arrows in Figure 7g–i). As can be seen in the enlarged figure, the glass fiber surface was rough, and there were many spherical holes (purple boxes in Figure 7i). The matrix surface between the fibers becomes rougher. The bond between the fiber and the resin is tight without cracks (purple dashed ellipse in Figure 7i). This no-crack bond indicates that cracks mainly propagate in the matrix, and the failure mode of the DCB specimen of GF/EP laminate composite is mostly in a cohesion failure mode [47,48]. The introduction of rigid nano-SiO_2_ improves the modulus of the matrix, thereby improving the stress transfer efficiency between the matrix and the fiber and avoiding interface adhesive failure with low energy consumption. At this time, cracks propagate in the matrix between the layers. The interlaminar toughness of the composite is mainly determined by the fracture toughness of the matrix, and the fracture toughness of the matrix is higher [49], so the *G_IC_*_,*Pro*_ value of the composite further increases to 1.42 kJ/m^2^, which is 88.2% higher than that of the unmodified composite.

## 4. Conclusions

This study experimentally investigated the fracture toughness of CTBN-toughened epoxy resin, CTBN + nano-SiO_2_ particle-toughened epoxy resin, and their GF/EP composite laminates. Mode I fracture toughness and toughening mechanism were identified:

1. The epoxy resin can be successfully toughened by CTBN. The epoxy resin’s mode I fracture toughness increases by 150% when CTBN concentration is 8 wt%, but its strength and modulus drop by 11.8% and 19.3%, respectively.
2. When flexible CTBN rubber particles and nano-SiO_2_ are used as synergistic toughening agents to toughen the epoxy resin base, when 8 wt% CTBN and 0.5 wt% nano-SiO_2_ are added to the resin, the fracture toughness of epoxy resin is increased by 215.8%. The tensile strength is only decreased by 2.3%, showing the best comprehensive performance.
3. The synergistic toughening effect of 8 wt% CTBN and 0.5 wt% nano-SiO_2_ increased the *G_IC_*_,*Pro*_ value of the GF/EP laminate composite by 86.8% and the interlaminate shear strength by 109.2%.
4. The cross-section analysis of GF/EP-laminated composites shows that the addition of flexible CTBN rubber particles and rigid nano-SiO_2_ makes the interface adhesive failure of GF/EP-laminated composites change to matrix cohesion failure. When cracks expand in the interlayer matrix, the cavitation of the two particles and the plastic deformation of the matrix is the toughening mechanism of the interlayer properties of the composite.

## Data Availability

Not applicable.
